# Zoonotic *Mycobacterium bovis*–induced Tuberculosis in Humans

**DOI:** 10.3201/eid1906.120543

**Published:** 2013-06

**Authors:** Borna Müller, Salome Dürr, Silvia Alonso, Jan Hattendorf, Cláudio J.M. Laisse, Sven D.C. Parsons, Paul D. van Helden, Jakob Zinsstag

**Affiliations:** Stellenbosch University, Cape Town, South Africa (B. Müller, S.D.C. Parsons, P.D. van Helden);; Swiss Tropical and Public Health Institute, Basel, Switzerland (B. Müller, J. Hattendorf, J. Zinsstag);; University of Basel, Basel (B. Müller, J. Hattendorf, J. Zinsstag);; University of Berne, Berne, Switzerland (S. Dürr);; Royal Veterinary College, Hertfordshire, UK (S. Alonso);; Veterinary Faculty, Eduardo Mondlane University, Maputo, Mozambique (C. J. M. Laisse)

**Keywords:** tuberculosis and other mycobateria, Mycobacterium bovis, zoonosis, bacteria, neglected tropical diseases, bovine tuberculosis, systematic review, TB, zoonotic TB, TB

## Abstract

We aimed to estimate the global occurrence of zoonotic tuberculosis (TB) caused by *Mycobacterium bovis* or *M. caprae* infections in humans by performing a multilingual, systematic review and analysis of relevant scientific literature of the last 2 decades. Although information from many parts of the world was not available, data from 61 countries suggested a low global disease incidence. In regions outside Africa included in this study, overall median proportions of zoonotic TB of ≤1.4% in connection with overall TB incidence rates ≤71/100,000 population/year suggested low incidence rates. For countries of Africa included in the study, we multiplied the observed median proportion of zoonotic TB cases of 2.8% with the continental average overall TB incidence rate of 264/100,000 population/year, which resulted in a crude estimate of 7 zoonotic TB cases/100,000 population/year. These generally low incidence rates notwithstanding, available data indicated substantial consequences of this disease for some population groups and settings.

Tuberculosis (TB) is among the most devastating human infectious diseases worldwide. An estimated 8.8 million new cases, a global average incidence rate of 128/100,000 population/year, and 1.5 million deaths were attributed to TB in 2010 (*1*). Human TB is caused principally by *M. tuberculosis*. The main causative agents of bovine \TB are *M. bovis* and, to a lesser extent, *M. caprae*; however, zoonotic transmission of these pathogens is well described and occurs primarily through close contact with infected cattle or consumption of contaminated animal products such as unpasteurized milk (*2*,*3*). TB cases caused by transmission of other mycobacteria from other animal reservoirs (e.g., wildlife) have been anecdotally reported (*4*,*5*). Globally, most cases of zoonotic TB are caused by *M. bovis*, and cattle are the major reservoir (*2*,*3*). Therefore, for the purpose of this study and the remainder of this report, we refer to zoonotic TB as TB in humans caused by *M. bovis* or *M. caprae*.

There is evidence to suggest that zoonotic TB accounted for a significant proportion of the TB cases in the Western world before the introduction of regular milk pasteurization programs (*6**,**7*). Currently, in high-income countries, bovine TB is well controlled or eliminated in most areas, and cases of zoonotic TB are rarely seen (*6*,*7*). However, reservoirs of TB in wildlife populations have been linked to the persistence or increase of the incidence of bovine TB in some countries, most notably the United Kingdom (UK) (*6*). The absence of zoonotic TB despite an upsurge in the incidence of bovine TB in the United Kingdom sparked a controversy over the large financial expenditures for disease control in cattle (*6*).

The situation may be fundamentally different in other regions. For example, in most countries in Africa, bovine TB is prevalent, but effective disease control, including regular milk pasteurization and slaughterhouse meat inspection, is largely absent (*2*,*3*). This situation is exacerbated by the presence of multiple additional risk factors such as human behavior and the high prevalence of HIV infections (*2*,*3*,*7*). Although HIV/AIDS is thought to facilitate transmission and progression to active disease of any form of TB, some studies showed a significantly increased proportion of *M. bovis* infections among HIV–co-infected TB patients compared with HIV-negative TB patients (*8*–*12*).

No assessment of the global consequences of zoonotic TB has yet been done. This may have been partially caused by the difficulty of differentiating TB caused by *M. tuberculosis* or *M. bovis*, which requires mycobacterial culture and the subsequent use of biochemical or molecular (e.g., genotyping) diagnostic methods. Therefore, in low-income countries, facilities to identify the causative agent of TB are largely absent (*2*,*3*,*7*). A previous comprehensive review on zoonotic TB was published 15 years ago with inferences based primarily on the presence of risk factors rather than the occurrence of actual cases (*2*). Since then, several studies of zoonotic TB in different parts of the world have been published, enabling a more detailed evaluation of the current importance of the disease. The current study was mandated by the World Health Organization (WHO) Foodborne Disease Burden Epidemiology Reference Group with the aim to determine, on the basis of previously published literature, the global occurrence of zoonotic TB and its contribution to the overall TB prevalence in affected settings.

## Materials and Methods

**Figure 1:**
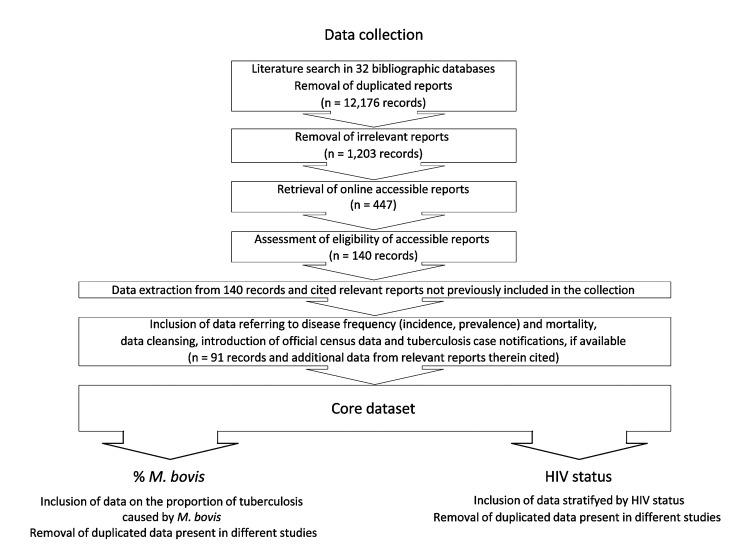
Selection procedure for reports included in this analysis. A list of all identified 1,203 potentially relevant reports and the core dataset is available as supplemental material (online Technical Appendix 2, wwwnc.cdc.gov/EID/article/19/6/12-0543-Techapp2.xlsx). TB, tuberculosis; *M. bovis*, *Mycobacterium bovis.*

A systematic multilingual literature search was performed according to international guidelines with certain modifications (http://www.cochrane-handbook.org/). Potentially relevant reports on putative zoonotic TB caused by *M. bovis* or *M. caprae* were identified by a search of 32 bibliographic databases by using a highly sensitive search syntax. All publications/reports documented in the various databases and published until March 2010 were considered (Table 1, Technical Appendix 1, and Technical Appendix 2). Reference Manager v11.0.1 bibliographic software was used to store and remove duplicated reports, leaving 12,176 records (Figure 1). Titles and abstracts of these reports were screened to remove studies unlikely to contain pertinent information. Altogether, 1,203 potentially relevant reports were identified (Technical Appendix 1, 2) of which 447 (37%) were available online and assessed for eligibility (Figure 1). 

**Table 1:** Number of countries within World Health Organization regions for which the respective data on zoonotic *Mycobacterium bovis*–induced TB in humans was obtained*

Variables	No. (%) of countries by WHO region

Africa	Americas	Europe	Eastern Mediterranean	Western Pacific
Total number of countries in WHO region†	46 (100.0)	35 (100.0)	53 (100.0)	22 (100.0)	27 (100.0)
Any data for countries of WHO region indicated‡	13 (28.3)	12 (34.3)	31 (58.5)	2 (9.1)	3 (11.1)
Nationwide data	0	10 (83.3)	30 (96.8)	0	2 (66.7)
Subnational data	13 (100.0)	5 (41.7)	11 (35.5)	2 (100.0)	2 (66.7)
Surveillance data	6 (46.2)	5 (41.7)	31 (100.0)	1 (50.0)	3 (100.0)
Convenience sample/unknown sampling strategy	10 (76.9)	11 (91.7)	6 (19.4)	1 (50.0)	2 (66.7)
Nationwide surveillance data	0	2 (16.7)	30 (96.8)	0	2 (66.7)
Molecular-based detection of *M. bovis*	10 (76.9)	7 (58.3)	9 (29.0)	2 (100.0)	3 (100.0)
Biochemical detection of *M. bovis*	6 (46.2)	3 (25.0)	4 (12.9)	0	1 (33.3)
Other detection method	1 (7.7)	3 (25.0)	3 (9.7)	0	1 (33.3)
Unknown detection method	0	10 (83.3)	30 (96.8)	0	2 (66.7)
Data on average yearly prevalence	9 (69.2)	6 (50.0)	5 (16.1)	0	2 (66.7)
Data on average yearly prevalence per 100,000 population	0	0	0	0	0
Data on average yearly incidence	6 (46.2)	10 (83.3)	31 (100.0)	1 (50.0)	3 (100.0)
Data on average yearly incidence per 100,000 population	2 (15.4)	7 (58.3)	30 (96.8)	1 (50.0)	2 (66.7)
Data on average yearly mortality	0	1 (8.3)	2 (6.5)	0	0
Data on average yearly mortality per 100,000 population	0	1 (8.3)	0	0	0
Data on proportion among all TB cases	13 (100.0)	12 (100.0)	30 (96.8)	2 (100.0)	3 (100.0)
Data on proportion among all TB deaths	0	1 (8.3)	0	0	0
Data on proportion of deaths among all TB cases	0	1 (8.3)	2 (6.5)	0	0

*TB, tuberculosis; WHO, World Health Organization; Freq, Number of countries represented by records reporting on the indicated variable; %, proportions related to the total number of countries in a given WHO region for which data were available (unless otherwise indicated). †No data were obtained for Southeast Asia. ‡Proportions relate to the total number of countries in the respective WHO region.

**Figure 2:**
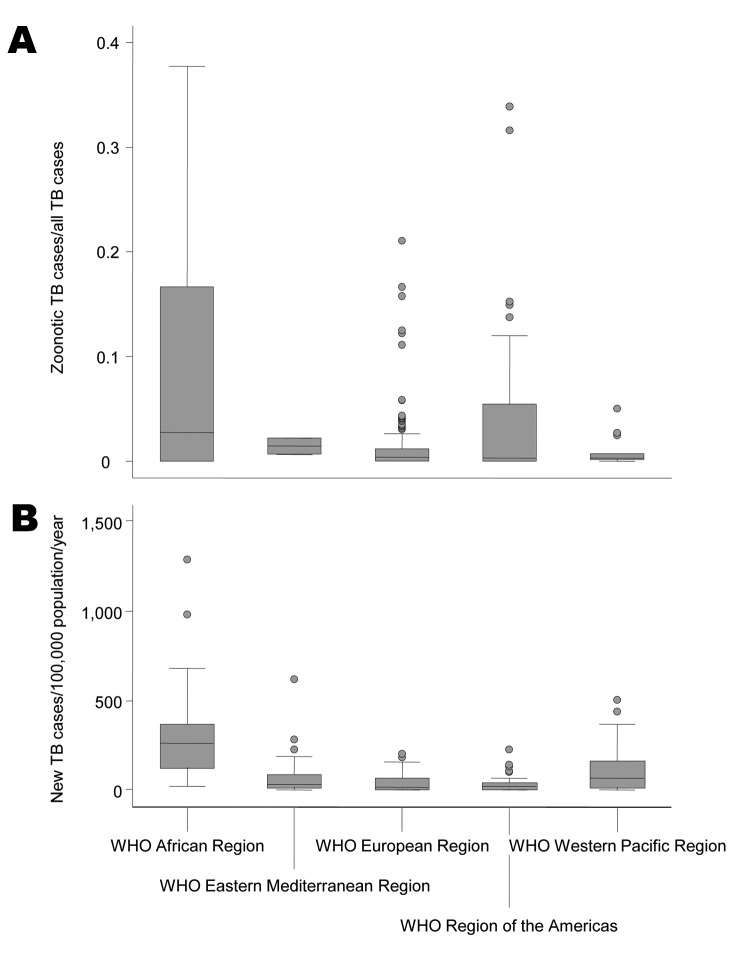
Proportion of zoonotic tuberculosis (TB) among all TB cases (A) and estimated overall TB incidence (B) stratified by World Health Organization (WHO) region. Overall TB incidence rates estimated for 2010 by WHO regions covered all countries of the respective regions. Numbers were obtained from WHO (*1*).

Eligible records (written in English, French, German, Spanish, or Portuguese) reported data for at least 50 persons tested on the frequency (prevalence, incidence) or death rate of putative zoonotic TB and contained data from no earlier than 1990. In connection with other ongoing studies, records reporting information on disease sequelae and transmission routes were also included. For 100 randomly selected reports of the 1,203 potentially relevant reports, availability and eligibility was assessed independently by 3 operators. Differently appraised records were reassessed, and the screening procedure was harmonized accordingly. The remaining records were randomly assigned to 1 of the 3 individual operators for further assessment; 140 records were considered eligible and subjected to data extraction. The data were stratified by multiple variables, if possible (e.g., country or province, HIV status). If any of the reports included referred to relevant external data or eligible reports that were not identified during the earlier steps of our literature search, the respective data were also included in this analysis. For 15 randomly selected reports of the 140 eligible records and additional data cited by or referring to these records, data extraction was performed independently by the 3 operators. Differently extracted data was reassessed and the extraction procedure was harmonized accordingly. The remaining records were randomly assigned to one of the 3 operators for data extraction. Of 140 eligible records, 91 reported or referred to data on disease frequency or mortality rates of putative zoonotic TB (Technical Appendix 1 Figure 2). If accessible, additional population estimates and official TB notifications were included in the database. This core dataset was used to analyze, by geographic region and country, the global occurrence of zoonotic TB in humans and its contribution to the overall TB prevalence in affected settings. A subset of data stratified by HIV status was used to assess a potential association between TB caused by *M. bovis* and HIV co-infection. For each of these analyses, duplicated data, present in >1 report was removed from the core dataset (Figure 1). Statistical analyses were performed in IC Stata 10.0 (StataCorp LP, College Station, TX, USA).

## Results

### Data Availability

Data obtained from eligible reports covered 5 of 6 WHO regions (Technical Appendix 1 Table 3, Figure 1), namely Africa, the Americas, Europe, the Eastern Mediterranean and the Western Pacific. No data were obtained for any country in Southeast Asia. With the exception of Europe, data were acquired for only a few countries of the regions represented in this analysis (Technical Appendix 1 Table 1). Notably, for the Eastern Mediterranean and the Western Pacific regions, respectively, data from only 2 and 3 countries were obtained. The considerable lack of data precluded a credible estimation of the global prevalence and incidence of zoonotic TB.

**Table 3:** Selected reports from the United States on zoonotic *Mycobacterium bovis*-induced TB in humans*

ID	Setting	Study period	Ethnicity or place of birth	No. cases†	No. tested‡	%§
46	41 states in the United States	1995–2005	Native American, non-Hispanic	0	243	0.0
			Asian, non-Hispanic	5	2,938	0.2
			Black, non-Hispanic	6	3,447	0.2
			Hispanic	147	2,724	5.4
			White, non-Hispanic	7	2,449	0.3
			Mexico-born	102	1,399	7.3
			Non-US/Mexico-born	15	4,907	0.3
			US-born	47	5,531	0.8
48	San Diego County, California	2001–2005	Hispanic	128	657	19.5
			White	3	154	1.9
			Asian	1	421	0.2
			Black	0	86	0.0
			Other	0	6	0.0
			Mexico-born	79	461	17.1
			The Philippines	0	248	0.0
			Non-US/Mexico/The Philippines-born	0	260	0.0
			US-born	53	355	14.9
164	San Diego County, California	1994–2003	Hispanic	156	531	29.4
			Non-Hispanic	11	564	2.0
			Mexico-born	93	374	24.9
			Non-US/Mexico-born	3	416	0.7
			US-born	71	305	23.3
155	New York, New York, USA	2001–2004	Mexico-born	20	155	12.9
			Non–Mexico-born	15	2925	0.5
233	San Diego County, California	1994–2000	Mexico-born	70	553	12.7
			The Philippines	1	423	0.2
			Non-US/Mexico/The Philippines–-born	2	403	0.5
			US-born	56	552	10.1

*TB. tuberculosis; ID, record identification number (online Technical Appendix 2, wwwnc.cdc.gov/EID/article/19/6/12-0543-Techapp2.xlsx).  †N number of zoonotic TB cases identified. ‡Number of TB case-patients tested for the presence of *M. bovis* infection.  § Proportion of zoonotic TB among all TB cases.

### Zoonotic TB in Africa

**Figure 3:**
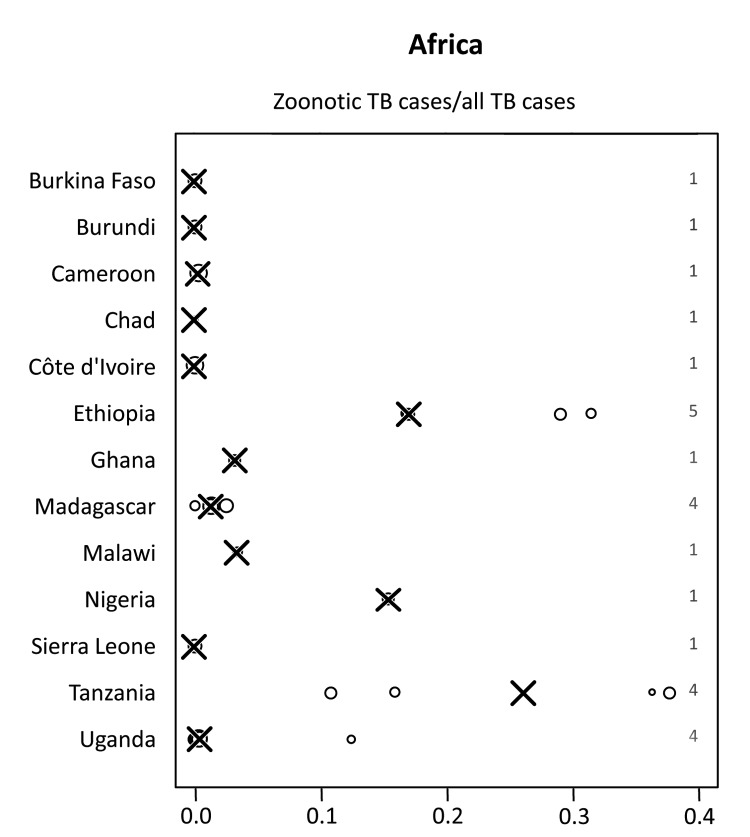
Proportion of zoonotic tuberculosis (TB) among all TB cases stratified by country: Africa. *x-*axis values are median proportions. Each circle represents a study with the circle diameter being proportional to the log_10_ of the number of isolates tested. A gray rhombus indicates that the number of samples tested was not reported or could not be inferred from the data available. The median proportion of all studies for a given country is indicated by X. Numbers on the right side of the figures indicate the number of studies included for any given country.

Among all studies included from Africa, a median of 2.8% (range 0%–37.7%) of all TB cases in humans were caused by *M. bovis* (Figure 2). In 10 of the 13 countries in Africa included in this study, median proportions of TB caused by *M. bovis* were below 3.5% and in 5 of these countries no cases were detected (Table 2; Figure 3). In contrast, for Ethiopia, Nigeria and Tanzania, respectively, the median proportion of TB cases caused by *M. bovis* was 17.0% (range: 16.7%–31.4%) (*13*–*15*), 15.4% (1 study available) (*16*) and 26.1% (range 10.8%–37.7%) (*17*–*19*). Percentages of ≈30% were reported in 4 regionally based studies in Tanzania and Ethiopia (Figure 3, Table 2) (*13*,*17*). However, many of these studies showed a low sample size, resulting in a high statistical error (Table 2). The lack of large-scale, population-based data did not allow for an identification of specific risk groups associated with *M. bovis* infections.

**Table 2:** Overview of selected reports from Africa on zoonotic *Mycobacterium bovis*–induced TB in humans*

ID	Country	Setting	Sampling	Detection	Years	*M. bovis*†	TB‡	%§	95% CI	Location
1131	Tanzania	NA	Conv.	Mol.	NA	20	53	37.7	24.8–52.1	EPTB
1131	Tanzania	Arusha region	Conv.	Mol.	1994	4	11	36.4	10.9–69.2	EPTB
1074	Ethiopia	Butajira health center, southeastern Ethiopia	System.	Mol.	2000/2001	11	35	31.4	16.9–49.3	EPTB
1074	Ethiopia	NA	Conv.	Biochem.	NA	14	48	29.2	17.0–44.1	PTB
15	Ethiopia	Felegehiwot hospital	Conv.	Biochem.	2007/2008	8	47	17.0	7.6–30.8	Both
65	Ethiopia	NA	Conv.	Biochem.	NA	7	42	16.7	7.0–31.4	Both
1074	Ethiopia	Fitche Hospital TB clinic	Conv.	Biochem.	2004/2005	7	42	16.7	7.0–31.4	Both
294	Tanzania	Arusha region and Southern Highlands	Surv.	Biochem.	1993–1996	7	44	15.9	6.6–30.1	Both
73	Nigeria	Jos	Conv.	Biochem.	NA	10	65	15.4	7.6–26.5	PTB
68	Uganda	Karamojo region	Conv.	Mol.	NA	3	24	12.5	2.7–32.4	EPTB
159	Tanzania	Three districts in Arusha region	Surv.	Biochem.	1999–2001	7	65	10.8	4.4–20.9	EPTB
459	Malawi	Blantyre, Queen Elizabeth Central Hospital	Conv.	Biochem.	NA	1	30	3.3	0.1–17.2	PTB
62	Ghana	Korle-Bu Teaching Hospital	Conv.	Biochem.	2003	2	64	3.1	0.4–10.8	PTB
464	Madagascar	Institut d'Hygiène Sociale, Antananarivo	Conv.	Mol.	1994	3	126	2.4	0.5–6.8	PTB
340	Madagascar	Antananarivo	Surv.	Other	1994/1995	2	156	1.3	0.2–4.6	EPTB
292	Madagascar	Antananarivo, Antsirabe, Fianarantsoa, and Mahajanga	Rand.	Mol.	1994/1995	4	316	1.3	0.3–3.2	PTB
243	Uganda	Kampala	Conv.	Biochem.	1995–1997	1	234	0.4	0.0–2.4	PTB
52	Uganda	Kampala, Rubaga division	Surv.	Mol.	2006	1	386	0.3	0.0–1.4	PTB
222	Cameroon	Ouest Province	Surv.	Mol.	1997/1998	1	455	0.2	0.0–1.2	PTB
80	Burkina Faso	Health centers in Ouagadougou and Bobo Dioulasso	Conv.	Mol.	2001	0	120	0.0	0.0–3.0¶	PTB
12	Burundi	Bujumbara, Bubanza Hospital	Conv.	Mol.	1987–1994	0	117	0.0	0.0–3.1¶	Both
165	Chad	Chari-Baguirmi	Conv.	Mol.	2002	0	10	0.0	0.0–30.8¶	Both
12	Côte d'Ivoire	TB and rural health centers in Côte d’Ivoire	Cluster	Mol.	1994–1996	0	320	0.0	0.0–1.1¶	PTB
464	Madagascar	Antananarivo prison	Conv.	Mol.	1994	0	36	0.0	0.0–9.7¶	PTB
12	Sierra Leone	Western Area and Kenema districts	Surv.	Mol.	2003/2004	0	97	0.0	0.0–3.7¶	PTB
32	Uganda	Mbarara district	Rand.	Mol.	2004/2005	0	70	0.0	0.0–5.1¶	Both

*TB, tuberculosis; ID, record identification number (online Technical Appendix 2, wwwnc.cdc.gov/EID/article/19/6/12-0543-Techapp2.xlsx); NA, not available; Conv., convenience sample/not specified; Mol., molecular-based detection method; EPTB, extrapulmonary TB; System, systematic sampling; Biochem, biochemical detection method; PTB, pulmonary TB; Surv.,surveillance data; Rand., random sampling; Cluster, cluster sampling. †Number of putative zoonotic cases of *Mycobacterium bovis*. ‡Total number of bacteriologically confirmed and characterized TB cases. §%, proportion of zoonotic TB among all TB cases. ¶One-sided 97.5% CIs are indicated.

### Zoonotic TB in the Americas

**Figure 4:**
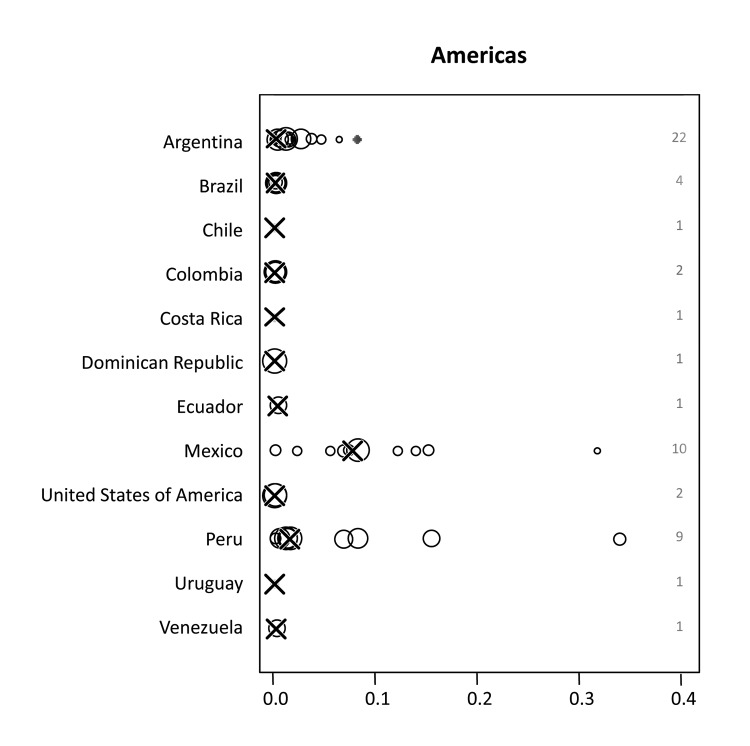
Proportion of zoonotic tuberculosis (TB) among all TB cases stratified by country: Americas. *x-*axis values are median proportions. Each circle represents a study with the circle diameter being proportional to the log_10_ of the number of isolates tested. A gray rhombus indicates that the number of samples tested was not reported or could not be inferred from the data available. The median proportion of all studies for a given country is indicated by X. Numbers on the right side of the figures indicate the number of studies included for any given country.

A median of 0.3% (range 0%–33.9%) of *M. bovis* infections among human TB cases was found for all reports included. For most countries, *M. bovis* accounted for a negligible percentage of the TB cases (Figure 2). Conversely, high proportions were reported for specific areas of Mexico and the United States (Figure 4). For Mexico, the median percentage of *M. bovis* cases was 7.6% (range 0%–31.6%); proportions >10% were detected in 3 independent studies (*20*–*22*). However, overall TB incidence in Mexico is relatively low, with a rate of 16/100,000 population/year (*1*). In the United States, TB caused by *M. bovis* is strongly linked to persons in Hispanic communities, mostly with origins in Mexico (Table 3). A study including data from 41 states of the United States suggested that ≈90% of all TB cases caused by *M. bovis* affect persons of Hispanic ethnicity (*8*). This association is attributed to the consumption of unpasteurized, contaminated cheese produced in Mexico. Moreover, when multivariate logistic regression analyses was used, several studies in the United States showed an independent association of TB caused by *M. bovis* with TB cases in children, HIV coinfection and extrapulmonary disease (*8*–*12*). Surveys in San Diego County, California indicated a steady increase in the incidence of TB caused by *M. bovis* and a decrease in TB incidence caused by *M. tuberculosis* infection (*9*,*12*). In this setting, the odds for TB patients infected with *M. bovis* to die during treatment was >2 times as high as for patients infected with *M. tuberculosis* (*9*,*10*,*12*). In San Diego County, during 1994–2003 and 2001–2005, respectively, *M. bovis* accounted for 25 and 19 deaths, corresponding to 27% and 17% of all TB deaths and a mortality rate of ≈0.1/100,000 population/year (*9*,*10*). The reasons for increased deaths among patients infected with zoonotic TB compared with those infected with *M. tuberculosis* remained unidentified, although health care inequality or treatment differences were stated as possible explanations (*9*). However, overall incidence rates of zoonotic TB in the United States are low, at a median of 0.7/100,000 population/year. Although zoonotic TB causes minor consequences of disease in the Americas, available data corroborate the finding that *M. bovis* infections can be a substantial cause of deaths from TB among humans in certain population groups and settings.

### Zoonotic TB in Europe

**Figure 5:**
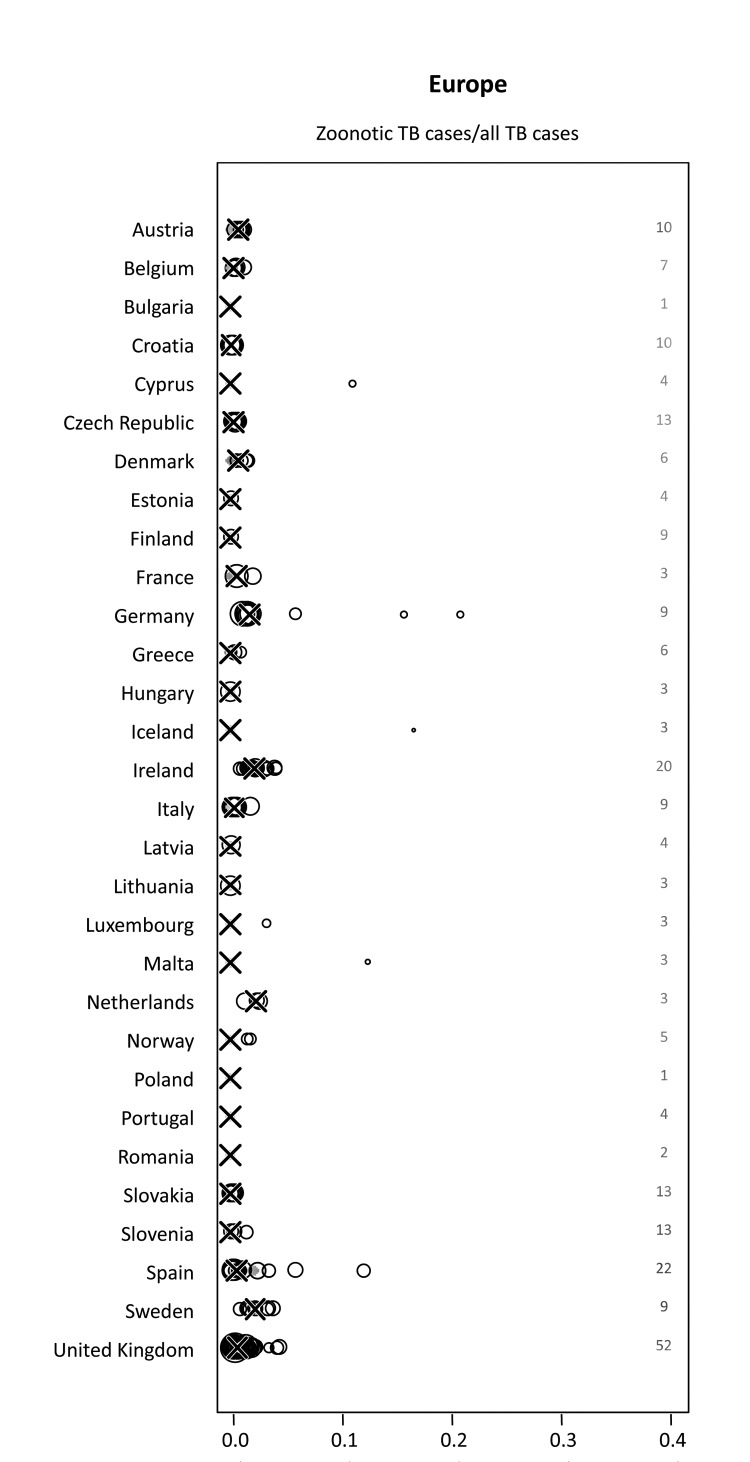
Proportion of zoonotic tuberculosis (TB) among all TB cases stratified by country: Europe. *x-*axis values are median proportions. Each circle represents a study with the circle diameter being proportional to the log_10_ of the number of isolates tested. A gray rhombus indicates that the number of samples tested was not reported or could not be inferred from the data available. The median proportion of all studies for a given country is indicated by X. Numbers on the right side of the figures indicate the number of studies included for any given country.

Studies from Austria, Germany, Greece, and Spain included in this analysis identified *M. caprae* as a causative agent of zoonotic TB in addition to *M. bovis* (*23*–*26*). Our analysis revealed a median proportion of 0.4% (range 0%–21.1%) of *M. bovis* or *M. caprae* infections among all bacteriologically confirmed TB cases reported. Median proportions for individual countries never reached rates >2.3%, although higher percentages were found for specific populations and settings (Figure 5). Three of 5 studies reporting proportions >10% were conducted in settings with very low incidences of human TB (<20 cases/year on a countrywide level) (*27*). In another hospital-based study in Germany, 4 *M. bovis* cases were identified among 19 TB cases with molecular speciation results, 2 of which probably represented disease caused by the treatment of urothelial carcinoma with *M. bovis* BCG (*28*). A study in Spain characterized the transmission of a multidrug-resistant strain of *M. bovis* as the cause of 2 nosocomial TB outbreaks that accounted for 12.2% of multidrug-resistant TB isolates (*29*). However, these cases did not represent cases of zoonotic TB, because transmission occurred from humans to humans. Reported incidence rates for TB caused by *M. bovis* or *M. caprae* for all studies included from European countries were <1/100,000 population/year if TB cases caused by multidrug-resistant strains of *M. bovis* in Spain were excluded (Technical Appendix 2). Moreover, available data suggested decreasing trends in the number of zoonotic TB cases over time (Technical Appendix 2).

### Zoonotic TB in the Eastern Mediterranean

**Figure 6:**
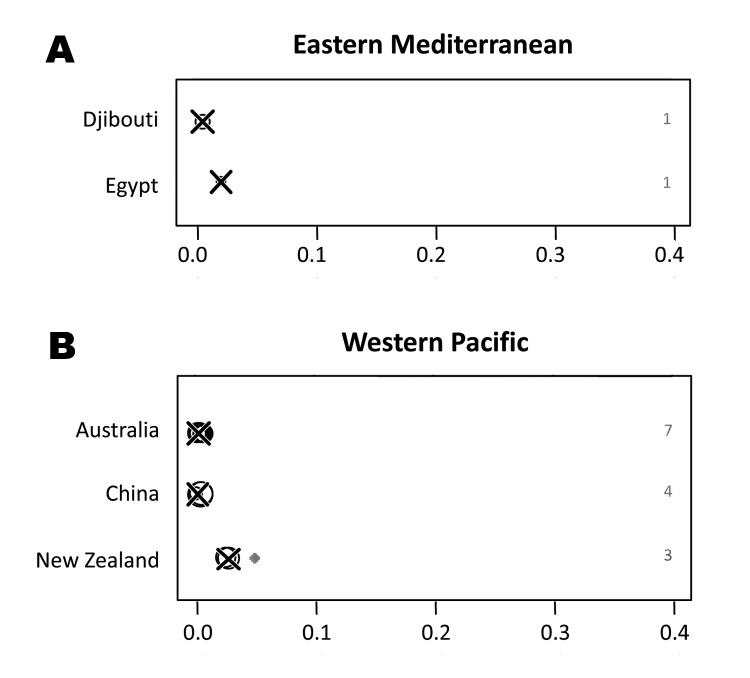
Proportion of zoonotic tuberculosis (TB) among all TB cases stratified by country: A) Eastern Mediterranean; B) Western Pacific. *x*-axis values are median proportions. Each circle represents a study with the circle diameter being proportional to the log_10_ of the number of isolates tested. A gray rhombus indicates that the number of samples tested was not reported or could not be inferred from the data available. The median proportion of all studies for a given country is indicated by X. Numbers on the right side of the figures indicate the number of studies included for any given country.

One study from the Suez Canal region of Egypt reported a rate of 2.2% (95% CI 0.1%–11.8%) for pulmonary TB cases caused by *M. bovis* (*30*). A second nationwide study in Djibouti detected 0.6% (95% CI 0.0%–3.5%) of the TB lymphadenitis cases for which samples were tested to be caused by *M. bovis* (*31*). No other studies were obtained for the WHO region of the Eastern Mediterranean. The proportion of zoonotic TB among all TB cases in the eastern Mediterranean is shown in Figure 6.

### Zoonotic TB in the Western Pacific

Data obtained from the Western Pacific region was from Australia, New Zealand, and parts of China only. For these settings, respectively, median proportions of *M. bovis* infections of 0.2% (range 0.1%–0.7%), 2.7% (range 2.4%–5%) and 0.2% (range 0%–0.5%) among all TB cases analyzed were observed, indicating that zoonotic TB had minor importance Median incidence rates were 0.03 (range 0.00–0.60) and 0.16 (range 0.11–0.27)/100,000 population/year for Australia and New Zealand, respectively. The infrequent occurrence of zoonotic TB in these settings notwithstanding, New Zealand showed a generally higher proportion and incidence rate for TB caused by *M. bovis* than did Australia (Figure 6). While a steadily increasing proportion of TB caused by *M. bovis* was observed in New Zealand, trends were decreasing in Australia (Technical Appendix 2).

### Influence of HIV Co-infection

Seven surveys covering Ethiopia, Tanzania, Argentina, Mexico, and different parts of the United States provided data for analysis of a potential association between HIV co-infection and zoonotic TB (Table 4) (*8*–*10*,*13*,*18*,*20*,*32*). Among these studies, only studies from the United States showed a significantly higher proportion of TB caused by *M. bovis* for HIV co-infected TB patients (Table 4). For studies from the USA, the relative risk for an infection with *M. bovis* among TB patients was 2.6–8.3 times higher in HIV–co-infected patients than in HIV-negative patients. No significant association between *M. bovis* infection and HIV status was identified in surveys in Africa or other countries of the Americas (Table 4).

**Table 4:** Available data on zoonotic *Mycobacterium bovis*-induced TB in humans, stratified by HIV-positive and -negative populations*

ID	Region	Country	HIV status	No. cases	No. tested	%	95% CI	RR
1074	Africa	Ethiopia	+	4	10	40.0	12.2–73.8	1.43
1074	Africa	Ethiopia	–	7	25	28.0	12.1–49.4	NA
1147	Africa	Tanzania	+	2	29	6.9	0.8–22.8	0.50
1147	Africa	Tanzania	–	5	36	13.9	4.7–29.5	NA
NA	Africa	Total	+	6	39	15.4	5.9–30.5	0.78
NA	Africa	Total	–	12	61	19.7	10.6–31.8	NA
57	Americas	Argentina	+	2	240	0.8	0.1–3.0	0.88
57	Americas	Argentina	–	95	10,000	1.0	0.8–1.2	NA
57	Americas	Argentina	+	8	1,391	0.6	0.2–1.1	2.66
57	Americas	Argentina	–	12	5,551	0.2	0.1–0.4	NA
39	Americas	Mexico	+	11	80	13.8	7.1–23.3	1.90
39	Americas	Mexico	–	6	83	7.2	2.7–15.1	NA
46	Americas	USA	+	21	891	2.4	1.5–3.6*	2.59
46	Americas	USA	–	59	6,472	0.9	0.7–1.2*	NA
48	Americas	USA	+	33	140	23.6	16.8–31.5*	3.24
48	Americas	USA	–	48	659	7.3	5.4–9.5*	NA
164	Americas	USA	+	39	64	60.9	47.9–72.9*	8.32
164	Americas	USA	–	35	478	7.3	5.2–10.0*	NA
NA	Americas	Total	+	114	2806	4.1	3.4–4.9*	3.70
NA	Americas	Total	–	255	23,243	1.1	1.0–1.2*	N/A

*TB, tuberculosis; ID, Record identification number (online Technical Appendix 2, wwwnc.cdc.gov/EID/article/19/6/12-0543-Techapp2.xlsx); Cases, number of zoonotic tuberculosis (TB) cases identified; Tested, number of TB cases tested for the presence of *M. bovis* infection; %, proportion of zoonotic TB among all TB cases; RR, relative risk ratio; NA, not available. Significantly higher proportion of *M. bovis* infection among TB patients with HIV coinfection.

## Discussion

Naturally, the occurrence of zoonotic TB is greatly dependent on the presence of TB in cattle. Information on the global distribution and prevalence of bovine TB is scarce, but available data suggest that TB in cattle is prevalent in virtually all major livestock-producing countries of the developing world and Africa, specifically (*2*,*3*,*7*). Disease control in cattle is largely absent in these regions. Consequently, the majority of the human population is at risk for exposure to bovine TB; and globally, the occurrence of zoonotic TB likely mirrors TB prevalence in cattle.

Our systematic literature search on the occurrence of zoonotic TB provided no data for the WHO region of Southeast Asia, including major cattle producing middle- and low-income countries (e.g., India, Bangladesh, Pakistan, Myanmar, Indonesia) (Technical Appendix 1) (*33*). Moreover, except for Europe, data were acquired for few countries of the regions represented in this analysis (Table 1) with some, in terms of livestock production, particularly relevant countries missing (e.g., Canada, Kenya, Russia, South Africa, Sudan, and Turkey) (*33*). Nationwide surveillance data were almost exclusively available for high-income countries that have programs in place for bovine TB control and regular milk pasteurization (Table 1). Although ample data were obtained for many low-risk, high-income countries, the lack of nationwide surveys in potential high-risk settings precluded a credible estimation of the global occurrence of zoonotic TB.

Recorded incidence rates for zoonotic TB in Europe, the United States, Australia, and New Zealand were consistently below 1/100,000 population/year (Technical Appendix 2). Incidence rates were unavailable for other countries. However, a crude estimate could be obtained by multiplying the observed country-specific median proportion of zoonotic TB by the respective overall TB incidence rates. This suggested incidence rates of zoonotic TB of ≈1/100,000 population/year or lower for all countries outside Africa included in this survey, except for the Republic of Djibouti that reported ≈4 zoonotic TB cases/100,000 population/year.

Africa is assumed to bear the highest consequences of zoonotic TB worldwide because of the frequent and concurrent presence of multiple risk factors (*2*,*3*,*7*). This is supported by the highest reported median proportions of TB caused by *M. bovis* in connection with the worldwide highest overall TB incidence rates (Figure 2). Given an observed median proportion of zoonotic TB of 2.8% and the continental average overall incidence of TB of 264/100,000 population/year (Figure 2), an incidence rate of 7 zoonotic TB cases/100,000 population/year could be estimated (≈20 times lower than the global overall TB incidence rate) (*1*). Presumably, this is an overestimate as high-risk livestock producing countries of Africa (e.g., Ethiopia, Madagascar, Nigeria, and Tanzania) were overrepresented in this analysis (*33*) and because studies reporting the presence of zoonotic TB can be expected to be overrepresented among the surveys included (see limitations of the study below). Together, this suggests a low incidence of zoonotic TB in Africa.

Individual studies from various regions reported high proportions of zoonotic TB for specific population groups and settings (Figures 3–6). For example, in the Hispanic community in the United States, zoonotic TB appeared to be a considerable proportion of all TB cases (Table 3) and was associated with the consumption of unpasteurized cheese from Mexico (*8*–*12*). The highest median proportions for TB caused by *M. bovis* were observed in countries in Africa: Ethiopia, Nigeria, and Tanzania (Figure 3). However, the specific populations affected and risk factors of zoonotic TB in these settings remain largely elusive. The highest proportions of zoonotic TB in Africa were reported in studies investigating cases of extra-pulmonary TB (Table 2). For example, of a total of 26 studies, 11 studies reported proportions of zoonotic TB >10%; 9 of those included cases of extrapulmonary TB; of the 15 studies reporting a proportion of zoonotic TB <3.3%, only 4 included extrapulmonary TB cases (Table 2). This may mirror a widely stated association of zoonotic TB with extra-pulmonary disease, perhaps reflecting the consumption of contaminated animal products as one of the main drivers of zoontic TB (*2**,**3**,**7*). It has been postulated that pastoralist and rural communities would be at greatest risk for zoonotic TB (*2*,*3*,*7*), but the lack of data for these population groups prevents confirmation of this assumption. Collected individual studies reporting high proportions of TB caused by *M. bovis* suggest pockets of zoonotic transmission of TB for specific population groups and settings.

Outside Africa, large proportions of *M. bovis* infections among TB case-patients have been found mostly in low-TB incidence settings such as Mexico. In Cyprus, Iceland, and Malta, proportions of TB caused by *M. bovis* of >10% were observed (Figure 5); however, these countries, nationwide, reported <20 human TB cases in the respective years. Similarly, a study in San Diego County, California, USA, showed an overall decreasing incidence of human TB while the incidence of zoonotic TB and therefore also the relative proportion of zoonotic TB has steadily increased (*9*,*12*). This suggests that commonly applied control efforts targeting *M. tuberculosis* transmission have little effect on the occurrence of zoonotic TB and probably reflects the distinct drivers of *M. tuberculosis* and zoonotic TB infection (e.g., aerosol transmission vs. foodborne infection) (*2*,*3*). Similarly, differences in the epidemiology of zoonotic TB are likely to exist between different regions. This could be mirrored by the association of zoonotic TB with HIV in the United States, but not in other areas included in this analysis (Table 4). Ascertaining the factors contributing to an association between HIV and zoonotic TB in some regions will require more in-depth research, thus eliminating potential confounders such as socioeconomic status, education level, national origin, and other factors.

The current study is affected by several biases. The sensitivity of this systematic literature review was affected by the selection of eligible reports and data extraction by a single operator. Also, only reports available online and written in English, French, German, Spanish, or Portuguese were included. Selected reports are biased toward surveys which identified or aimed to identify TB cases caused by *M. bovis*, possibly resulting in an overestimation of the proportion of zoonotic TB cases. Data from low-income countries included in this study were rarely comparable and not representative of the respective nationwide populations. Nonetheless, it seems unlikely that our conclusions were fundamentally affected by these biases. Lastly, our results are influenced by the technical constraints of the studies included. Specifically, biochemical methods may be relatively unreliable for the identification of *M. bovis* or *M. caprae* strains and routine culture methods for *M. tuberculosis* are suboptimal to detect strains of *M. bovis* (*2*,*3*). Thus, TB cases caused by *M. bovis* may be systematically underreported.

Reports published after the completion of this systematic review revealed information from countries not covered by this study. A study from Bangladesh analyzed isolates from 350 TB patients but did not identify any infections by *M. bovis* (*34*). In a study from Bamako, Mali, 0.8% of TB cases analyzed were caused by *M. bovis* (*35*). In Turkey and the West Bank, Palestine, respectively, 5.3% and 6.5% of clinical TB cases analyzed were caused by *M. bovis* (*36*,*37*); however, zoonotic TB can be considered rare in these areas, given the low overall incidence rates of TB of 28 and 0.7/100,000 population/year (*1*,*38*). Together, available data suggest a minor global importance of zoonotic TB. However, pockets of more frequent zoonotic transmission of TB seem to be present in certain population groups. More research is needed to identify the main transmission drivers in these areas.

Technical Appendix 1 All searched bibliographic databases; search terms used to identify potentially relevant reports; member states of the World Health Organization regions; and heat map of the number of studies included in this analysis for each country.

Technical Appendix 2 Database of all identified 1,203 potentially relevant reports; core dataset extracted from eligible records including data on disease frequency and mortality; and variable names used for the core dataset.
