# Supplementary material for: Zoonotic Mycobacterium bovis–induced Tuberculosis in Humans
Source: Emerg Infect Dis. 2013 Jun;19(6):899–908. doi: 10.3201/eid1906.120543 (PMC4816377; doi:10.3201/eid1906.120543)
Supplement: Technical Appendix 1 — All searched bibliographic databases; search terms used to identify potentially relevant reports; member states of the World Health Organization regions; and heat map of the number of studies included in this analysis for each country. [file 12-0543-Techapp-s1.pdf]

Publisher: CDC; Journal: Emerging Infectious Diseases
Article Type: Synopsis; Volume: 19; Issue: 6; Year: 2013; Article ID: 12-0543
DOI: 10.3201/eid1906.120543; TOC Head: Synopsis
Article DOI: http://dx.doi.org/10.3201/eid1906.120543

Zoonotic *Mycobacterium bovis*–induced Tuberculosis in Humans

Technical Appendix 1

Technical Appendix 1 Table 1. Searched bibliographic databases

Database	URL	No. articles	Search syntax	Search date	Region	Comments
PubMed/MEDLINE	http://www.ncbi.nlm.nih.gov/pubmed/	7767	complete	21/04/2010	International	
ISI Web of Knowledge	http://isiwebofknowledge.com/	8324	complete	22/04/2010	International	
Popline	http://www.popline.org/	70	complete	21/04/2010	International	
CAB Abstracts and Global Health	http://www.cabdirect.org/	27	complete	06/05/2010	International	
ProMed	http://www.promedmail.org	241	modified	28/04/2010	International	
The Cochrane Library	http://www.thecochranelibrary.com	11	complete	21/04/2010	International	
BIOLINE	http://www.bioline.org.br	32	modified	29/04/2010	International	
WHOLIS	http://www.bireme.br	17	complete	06/05/2010	International	
Health Information Locator	http://www.bireme.br	3	complete	06/05/2010	International	
Institute of Tropical Medicine, Antwerp, Belgium	http://lib.itg.be:8000/webspirs/start.ws	14	complete	05/05/2010	International	
King's Fund Information & Library Service	http://www.kingsfund.org.uk/library/ http://kingsfund.koha-ptfs.eu/	0	modified	21/04/2010	International	Grey literature
African Journals Online	http://ajol.info/	691	modified	05/05/2010	Africa	
African Index Medicus	http://indexmedicus.afro.who.int/	0	modified	21/04/2010	Africa	
Afro Library	http://afrolib.afro.who.int/	0	modified	21/04/2010	Africa	
Latin American and Caribbean Health Science	http://www.bireme.br	496	complete	29/04/2010	Latin America	
MedCarib	http://www.bireme.br	24	complete	06/05/2010	Caribbean	
REPIDISCA	http://www.bireme.br	1	complete	06/05/2010	Latin America and Caribbean	
PAHO	http://www.bireme.br	35	complete	06/05/2010	Pan-America	
IBECS	http://www.bireme.br	94	complete	06/05/2010	Spanish literature	
CUIDEN	http://www.index-f.com/	2	modified	05/05/2010	Spanish literature	
HELLIS	http://www.hellis.org/	0	modified	21/04/2010	Asia	
Index Medicus for the South-East Asia Region	http://www.who.int/library/databases/searo/en/index.html	82	modified	29/04/2010	South-East Asia	
Western Pacific Region Index Medicus	http://www.who.int/library/databases/wpro/en/index.html	71	modified	29/04/2010	Western Pacific	
Indian Medlars Center - IndMed	http://indmed.nic.in/	22	modified	03/05/2010	Indian literature	
KoreaMed	http://www.koreamed.org/SearchBasic.php	79	modified	03/05/2010	Korean literature	
Japan Science and Technology Information Aggregator	http://www.jstage.jst.go.jp/browse/	309	modified	05/05/2010	Japanese literature	
Health Research and Development Information Network	http://www.herdin.ph/	4	modified	03/05/2010	Philippine literature	
Index Medicus for the Eastern Mediterranean Region	http://www.who.int/library/databases/emro/en/index.html	8	modified	28/04/2010	Eastern Mediterranean	
Panteleimon	www.panteleimon.org/maine.php3	8	modified	03/05/2010	Russian literature	
l'Ecole Nationale de la Santé Publique	http://www.bdsp.ehesp.fr/Base/	48	complete	05/05/2010	French literature	Additional search in French
La Bibliothèque de Santé	http://www.santetropicale.com/resum	1	modified	05/05/2010	Tropics,	Additional

Database	URL	No. articles	Search syntax	Search date	Region	Comments
Tropicale	e/catalogue.asp				French literature	search in French
System for Information on Grey Literature in Europe	opensigle.inist.fr	4	complete	06/05/2010	Europe	Grey literature
Total	N/A	12176	N/A	N/A	N/A	

Search syntax: Depending on whether or not the search engine did allow for the use of Boolean operators a complete or modified search syntax was used (Appendix Table 2).
Total: Sum of records after removal of identified duplicates.

Technical Appendix 1 Table 2. Search terms used to identify potentially relevant reports

A:	B:
"bovine tuberculosis"	"zoonotic"
"bovine TB"	"zoonosis"
"BTB"	"patient"
"zoonotic tuberculosis"	"patients"
"zoonotic TB"	"cohort"
"animal tuberculosis"	"population"
"animal TB"	"person"
"Mycobacterium bovis"	"persons"
"M. bovis"	"people"
"scrofula"	"child"
"Pott's disease"	"children"
"lupus vulgaris"	"adult"
	"adults"
	"woman"
	"women"
	"man"
	"men"
	"human"
	"humans"

Potentially relevant reports were identified using the search tools of the respective bibliographic databases (Technical Appendix Table 1). The search syntax was adapted to the different search tools. At least one of the search terms under A had to be present in connection with at least one of the terms under B. The most sensitive search settings had been applied. If the respective search tool did not allow for the use of Boolean operators, all reports that were retrieved for any of the search terms under A were used. Search terms were translated into French for searches in French literature databases (Technical Appendix Table 1).

Technical Appendix 1 Table 3. Countries of all World Health Orgazinzation regions

Countries in the WHO African Region	Countries in the WHO Region of the Americas	Countries in WHO South-East Asia Region	Countries in the WHO European Region	Countries in the WHO Eastern Mediterranean Region	Countries in the WHO Western Pacific Region
Algeria	Antigua and Barbuda	Bangladesh	Albania	Afghanistan	Australia
Angola	Argentina	Bhutan	Andorra	Bahrain	Brunei Darussalam
Benin	Bahamas	Democratic People's Republic of Korea	Armenia	Djibouti	Cambodia
Botswana	Barbados	India	Austria	Egypt	China
Burkina Faso	Belize	Indonesia	Azerbaijan	Iran (Islamic Republic of)	Cook Islands
Burundi	Bolivia (Plurinational State of)	Maldives	Belarus	Iraq	Fiji
Cameroon	Brazil	Myanmar	Belgium	Jordan	Japan
Cape Verde	Canada	Nepal	Bosnia and Herzegovina	Kuwait	Kiribati
Central African Republic	Chile	Sri Lanka	Bulgaria	Lebanon	Lao People's Democratic Republic
Chad	Colombia	Thailand	Croatia	Libya	Malaysia
Comoros	Costa Rica	Timor-Leste	Cyprus	Morocco	Marshall Islands

Countries in the WHO African Region	Countries in the WHO Region of the Americas	Countries in WHO South-East Asia Region	Countries in the WHO European Region	Countries in the WHO Eastern Mediterranean Region	Countries in the WHO Western Pacific Region
Congo	Cuba		Czech Republic	Oman	Micronesia (Federated States of)
Côte d'Ivoire	Dominica		Denmark	Pakistan	Mongolia
Democratic Republic of the Congo	Dominican Republic		Estonia	Qatar	Nauru
Equatorial Guinea	Ecuador		Finland	Saudi Arabia	New Zealand
Eritrea	El Salvador		France	Somalia	Niue
Ethiopia	Grenada		Georgia	South Sudan	Palau
Gabon	Guatemala		Germany	Sudan	Papua New Guinea
Gambia	Guyana		Greece	Syrian Arab Republic	Philippines
Ghana	Haiti		Hungary	Tunisia	Republic of Korea
Guinea	Honduras		Iceland	United Arab Emirates	Samoa
Guinea-Bissau	Jamaica		Ireland	Yemen	Singapore
Kenya	Mexico		Israel		Solomon Islands
Lesotho	Nicaragua		Italy		Tonga
Liberia	Panama		Kazakhstan		Tuvalu
Madagascar	Paraguay		Kyrgyzstan		Vanuatu
Malawi	Peru		Latvia		Viet Nam
Mali	Saint Kitts and Nevis		Lithuania		
Mauritania	Saint Lucia		Luxembourg		
Mauritius	Saint Vincent and the Grenadines		Malta		
Mozambique	Suriname		Monaco		
Namibia	Trinidad and Tobago		Montenegro		
Niger	United States of America		Netherlands		
Nigeria	Uruguay		Norway		
Rwanda	Venezuela (Bolivarian Republic of)		Poland		
Sao Tome and Principe			Portugal		
Senegal			Republic of Moldova		
Seychelles			Romania		
Sierra Leone			Russian Federation		
South Africa			San Marino		
Swaziland			Serbia		
Togo			Slovakia		
Uganda			Slovenia		
United Republic of Tanzania			Spain		
Zambia			Sweden		
Zimbabwe			Switzerland		
			Tajikistan		
			The former Yugoslav Republic of Macedonia		
			Turkey		
			Turkmenistan		
			Ukraine		
			United Kingdom		
			Uzbekistan		

Publisher: CDC; Journal: Emerging Infectious Diseases
Article Type: Synopsis; Volume: 19; Issue: 6; Year: 2013; Article ID: 12-0543
DOI: 10.3201/eid1906.120543; TOC Head: Synopsis

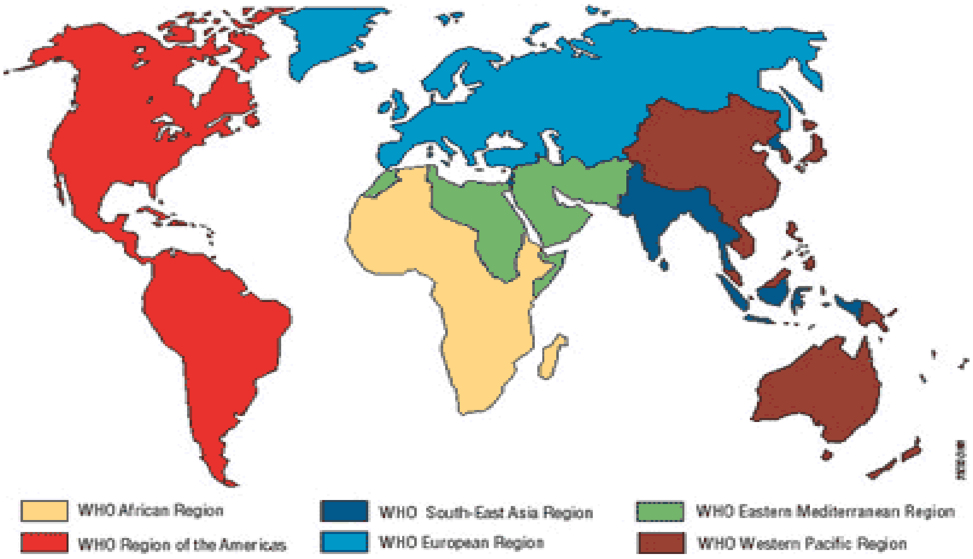

Technical Appendix 1 Figure 1. World Health Organization regions.

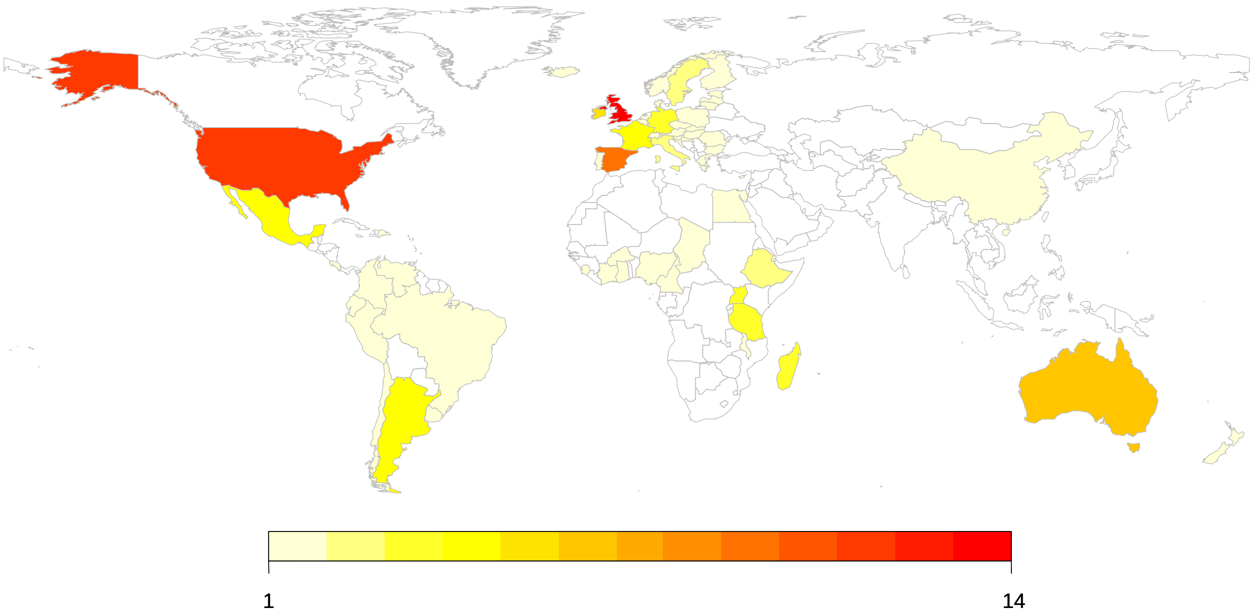

Technical Appendix 1 Figure 2. Heat map of the number of records used by country for the analyses herein performed.
